# Supplementary material for: The Adenoviral E1B-55k Protein Present in HEK293 Cells Mediates Abnormal Accumulation of Key WNT Signaling Proteins in Large Cytoplasmic Aggregates
Source: Genes (Basel). 2021 Nov 29;12(12):1920. doi: 10.3390/genes12121920 (PMC8701144; doi:10.3390/genes12121920)
Supplement: Supplementary file 1 [file genes-12-01920-s001.zip › Figure_S1.pdf]

Supplementary Figure S1. Localization of key WNT/β-catenin signaling components in the HEK293T cell line.

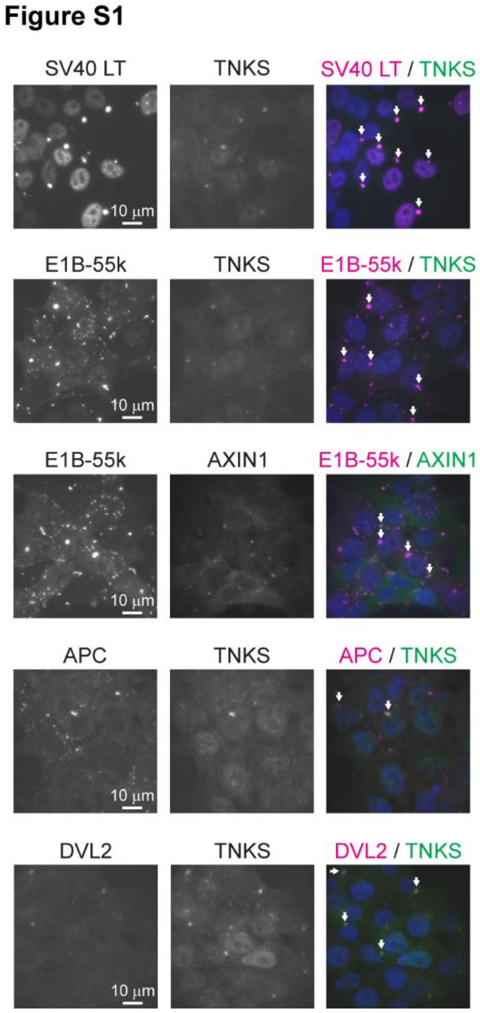

Figure S1. Localization of key WNT/β-catenin signaling components in the HEK293T cell line.

IF analysis (WF) of the localization of SV40-Large T antigen ((SV40 LT) not present in HEK293 cells), TNKS, E1B-55k, AXIN1, APC and DVL2 as indicated. In the merged color images co-localization is indicated with arrows and nuclear counterstaining is shown in blue.
