# Supplementary material for: The Adenoviral E1B-55k Protein Present in HEK293 Cells Mediates Abnormal Accumulation of Key WNT Signaling Proteins in Large Cytoplasmic Aggregates
Source: Genes (Basel). 2021 Nov 29;12(12):1920. doi: 10.3390/genes12121920 (PMC8701144; doi:10.3390/genes12121920)
Supplement: Supplementary file 1 [file genes-12-01920-s001.zip › Figure_S2.pdf]

Supplementary Figure S2. Measurement of E1B-55k protein layer thickness.

Figure S2

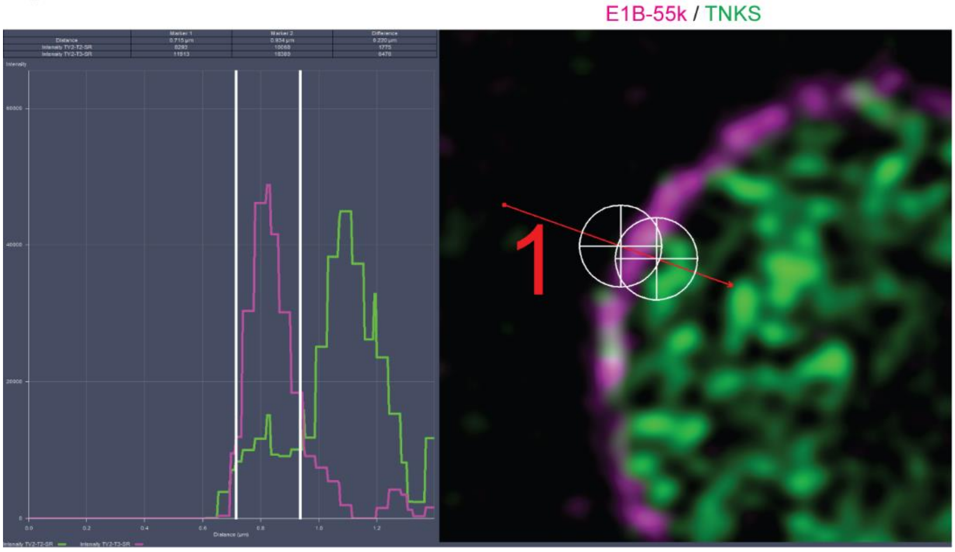

Figure S2. Measurement of E1B-55k protein layer thickness in representative TNKS aggregate.

The thickness of the E1B-55k protein layer was measured in a SIM image slice using the ZEN black software measurement tool. The histogram to the left shows the intensity plot of the E1B-55k (magenta) and TNKS (green) IF staining over the area covered by the red line in the image to the right. The white vertical lines in the histogram correspond to the center position of the aim crosses in the image. The measured distance between the lines and hence the thickness of the E1B-55k protein layer at the indicated position is 220 nm.
