# Supplementary material for: The Adenoviral E1B-55k Protein Present in HEK293 Cells Mediates Abnormal Accumulation of Key WNT Signaling Proteins in Large Cytoplasmic Aggregates
Source: Genes (Basel). 2021 Nov 29;12(12):1920. doi: 10.3390/genes12121920 (PMC8701144; doi:10.3390/genes12121920)
Supplement: Supplementary file 1 [file genes-12-01920-s001.zip › Figure_S4.pdf]

Supplementary Figure S4. In HEK293 cells with reduced E1B-55k protein levels the distribution of AXIN1, APC and DVL2 is changed from accumulation in aggregates to a more uniform cytoplasmic distribution.

Figure S4

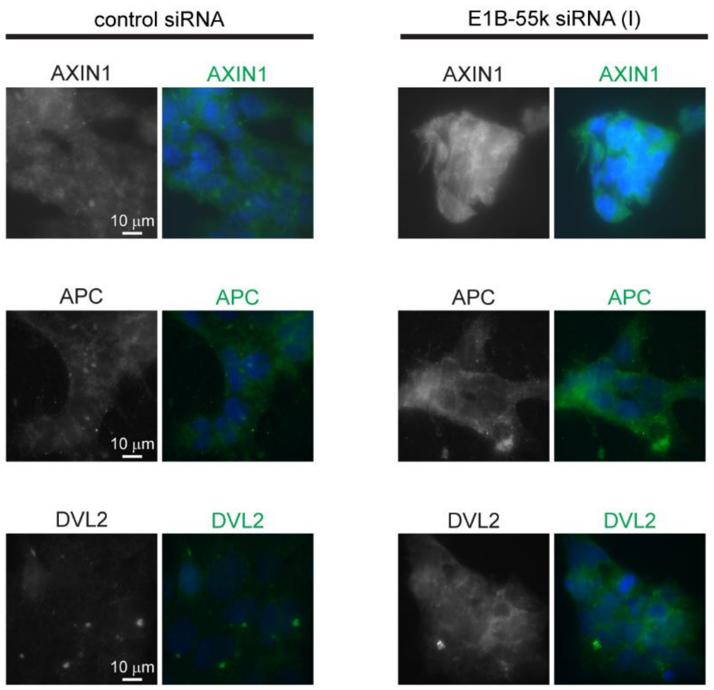

Figure S4. In HEK293 cells with reduced E1B-55k protein levels the distribution of AXIN1, APC and DVL2 is changed from accumulation in aggregates to a more uniform cytoplasmic distribution.

IF analysis (laser WF) of the localization of AXIN1, APC and DVL2 in HEK293 cells treated with control or E1B-55k siRNA. In the merged color images nuclear counterstaining is shown in blue.
