# Supplementary material for: The Adenoviral E1B-55k Protein Present in HEK293 Cells Mediates Abnormal Accumulation of Key WNT Signaling Proteins in Large Cytoplasmic Aggregates
Source: Genes (Basel). 2021 Nov 29;12(12):1920. doi: 10.3390/genes12121920 (PMC8701144; doi:10.3390/genes12121920)
Supplement: Supplementary file 1 [file genes-12-01920-s001.zip › Table_S1.pdf]

Supplementary Table S1. E1B-55k interacting partners detected by Co-IP

Experiment 1	Experiment 2	Identified in both experiments
RAD50	*RAD50*	*RAD50*
MRE11	*USP9X*	*USP9X*
USP9X	*MRE11*	*MRE11*
TP53	*TP53*	*TP53*
SDCCAG3	*PRRC2B*	*PRRC2B*
KRT78	*PHLDB2*	*PHLDB2*
PRRC2B	*NBN*	*NBN*
ATAD3A	SNX9	*HADHA*
PHLDB2	YTHDC2	*WAPL*
ARG1	*HADHA*	*TRIM27*
NBN	APC	*SDCCAG3*
TGM3	RAB11FIP2	*BAG2*
CASP14	*WAPL*	*IRS4*
MEIOC	DSG1	*LAMB3*
TNRC6A	TCOF1	*TNRC6A*
BAG2	*TRIM27*	*CUL9*
ZNF318	*SDCCAG3*	*ZNF318*
PRC1	*BAG2*	*CASP14*
TNRC6B	*IRS4*	*MYO1B*
TRAF7	TCF25	*MEIOC*
LAMB3	*LAMB3*	*ERH*
LMO7	TRIM28	
CUL7	CLASP1	
AZGP1	*TNRC6A*	
ERH	HAL	
KPRP	PKP4	
MYO1B	ZSWIM8	
AKAP8	*CUL9*	
ENO1	*ZNF318*	
GGCT	COIL	
CEP131	SNX33	
POF1B	FYCO1	
CORO1C	WASHC2A	
TRIM27	PCM1	
HNRNPF	GTF3C3	
CALML5	AGTPBP1	
KRT6C	TNRC6C	
DSC3	LAMA3	
GIGYF1	AGRN	
CUL9	LAMB1	
MYO1C	*CASP14*	
MYH10	RPS24	
ALOX12B	RPL4	
HADHA	SBSN	
SERPINB12	ITIH2	
AUNIP	VTN	
LIMA1	*MYO1B*	
WAPL	FLNA	
FLG	CARD10	
DLC1	*MEIOC*	
KIF14	SLC25A11	
DDX17	AMER1	
EIF4E2	TJP2	
UNC13C	BAIAP2L1	
IRS4	ZSCAN20	
BCLAF1	FAM83B	
IGHG4	SBNO1	
	AHDC1	
	RPL35	
	ERH	

Proteins in experiment 1 and 2 are sorted according to their "Mascot Score". Proteins detected in both experiments are in italic and previously identified E1B-55k interacting proteins are underlined. Further details can be found in Supplementary Table S2 and S3.
