# Supplementary figures and images for: The Adenoviral E1B-55k Protein Present in HEK293 Cells Mediates Abnormal Accumulation of Key WNT Signaling Proteins in Large Cytoplasmic Aggregates
Source: Genes (Basel). 2021 Nov 29;12(12):1920. doi: 10.3390/genes12121920 (PMC8701144; doi:10.3390/genes12121920)

**Supplementary Figure S3. Western blot analysis of E1B-55k protein levels in siRNA treated HEK293 cells.**

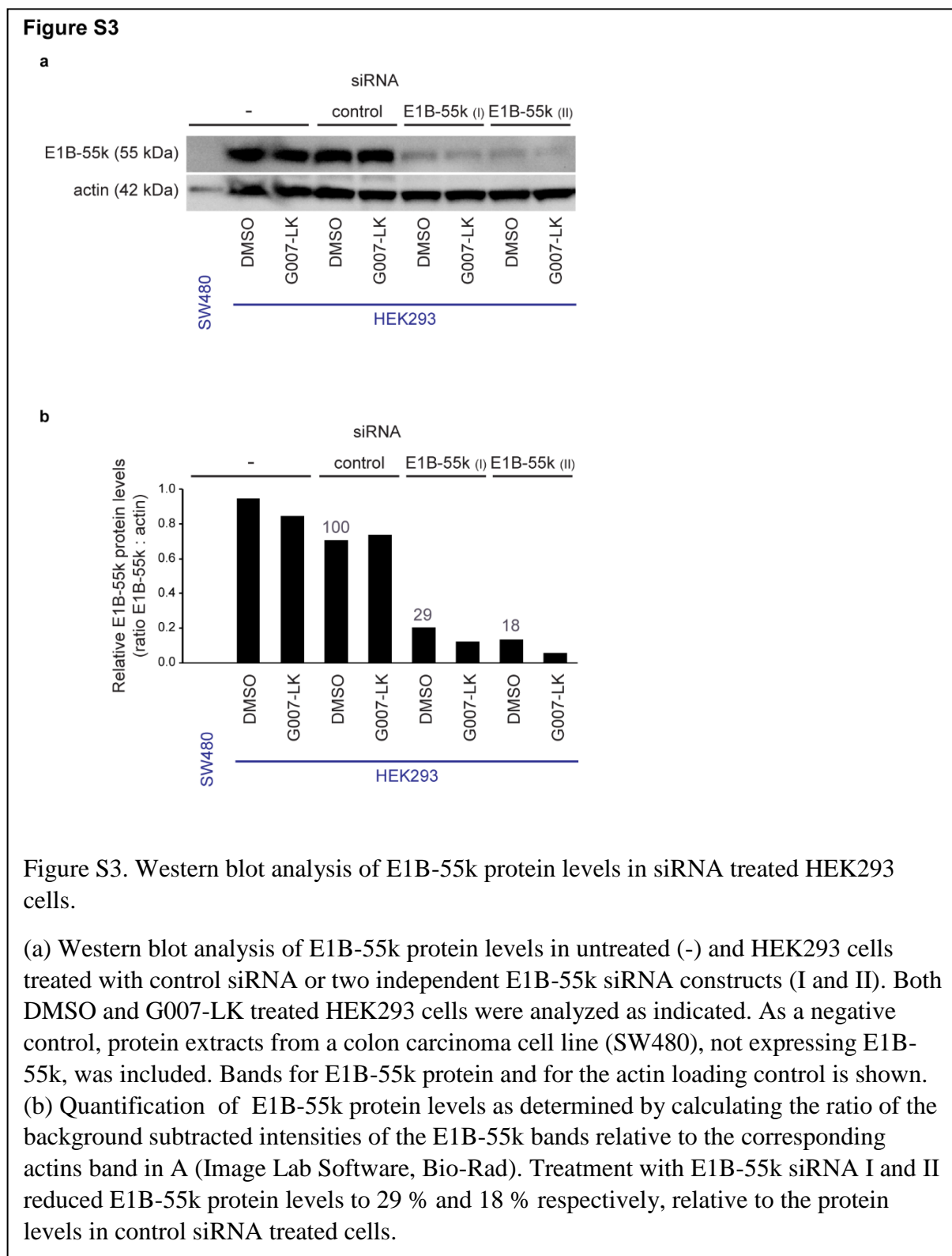

Supplement: Supplementary file 1 [file genes-12-01920-s001.zip › Figure_S3.pdf]
